# Economic Impact of Asbestos in Colombia: Estimation of the Direct and Societal Costs of Lung Cancer and Mesothelioma in 2022

**DOI:** 10.3390/ijerph23081044

**Published:** 2026-08-11

**Authors:** Guillermo Villamizar, Tim Driscoll, Shane McArdle, Arthur Frank

**Affiliations:** 1 Colombia Asbestos Free Foundation (FundClas), Bogotá 111211, Colombia; 2 Asbestos and Dust Diseases Research Institute (ADDRI), Sydney, NSW 2139, Australia; 3 Sydney School of Public Health, University of Sydney, Sydney, NSW 2050, Australia; 4 Dornsife School of Public Health, Drexel University, Philadelphia, PA 19104, USA

**Keywords:** asbestos, mesothelioma, lung cancer, economic burden, cost of illness, occupational cancer, public health, Colombia

## Abstract

**Highlights:**

**Public Health Relevance**

- Asbestos-related diseases continue to generate substantial health and economic consequences in Colombia despite the national asbestos ban enacted in 2019. The long latency period of asbestos-related malignancies means that historical exposures will continue producing disease burden for decades.
- Quantifying the economic burden of asbestos-related diseases provides essential evidence for public health decision-making and highlights the economic value of prevention, surveillance, and risk reduction strategies.

**Public Health Significance**

- This study provides the first comprehensive estimate of the direct and societal costs attributable to asbestos-related lung cancer and mesothelioma in Colombia.
- The findings demonstrate that the economic burden extends far beyond healthcare expenditures when productivity losses and quality-of-life impacts are considered.

**Public Health Implications**

- Strengthened surveillance systems, disease registries, and long-term management of installed asbestos are needed to reduce future disease burden.
- Economic burden estimates can support evidence-based decision-making for prevention, risk mitigation, and public health planning in post-ban settings.

**Abstract:**

Asbestos exposure remains an important cause of lung cancer and mesothelioma worldwide, and the long latency of asbestos-related diseases means that health and economic consequences persist even after asbestos bans. In Colombia, the economic burden attributable to historical asbestos exposure has not been comprehensively quantified. This study estimated the economic burden attributable to asbestos exposure in Colombia in 2022 using a prevalence-based cost-of-illness framework from a societal perspective. Direct medical costs were derived from a national lung cancer cost study and updated to 2022 values. Epidemiological inputs were obtained from the Global Burden of Disease (GBD) database. Mesothelioma was considered predominantly attributable to asbestos exposure, while lung cancer was adjusted using population-attributable fractions and an alternative 1:3 mesothelioma-to-lung-cancer ratio scenario. Indirect costs were estimated using the human capital approach, and disability-adjusted life year (DALY) losses were monetized using alternative willingness-to-pay thresholds. Colombia was estimated to have 150 prevalent mesothelioma cases and between 389 and 450 asbestos-attributable lung cancer cases in 2022. Direct costs ranged from USD 6.5 to 10.7 million annually, increasing to USD 16.7–23.1 million when productivity losses were included. Expanded societal valuation scenarios ranged from USD 71 million to more than USD 180 million annually. Asbestos-related malignancies continue to impose a substantial economic burden in Colombia and support the need for strengthened surveillance, long-term management of installed asbestos, and policies aimed at reducing future asbestos-related disease burden.

## 1. Introduction

Asbestos has historically been one of the most widely used industrial materials worldwide due to its heat resistance, durability, and versatility in industrial applications [1,2]. However, since the mid-twentieth century, substantial scientific evidence has established its carcinogenic properties, particularly in relation to pleural mesothelioma and lung cancer [3,4]. The International Agency for Research on Cancer (IARC) classified all forms of asbestos as Group 1 carcinogens, confirming a causal association with mesothelioma, lung cancer, and other malignancies [5], and no threshold below which exposure can be considered risk-free has been established [6,7,8,9].

A defining characteristic of asbestos-related diseases is their prolonged latency period, often exceeding 20 to 40 years between initial exposure and clinical manifestation [10]. Consequently, even in countries where asbestos has been legally banned for much longer than in Colombia, the health consequences of historical exposure persist for decades [11,12]. This delayed disease burden generates not only substantial morbidity and mortality but also long-term economic consequences for healthcare systems and society [13].

Global epidemiological models, including the Global Burden of Disease (GBD) study, estimate the burden attributable to occupational asbestos exposure across multiple cancer outcomes [14]. However, in the present study, we focus specifically on lung cancer and mesothelioma. This restriction does not imply that these are the only malignancies associated with asbestos exposure [5]. Rather, they were selected because they represent the most epidemiologically consistent asbestos-related outcomes and, critically, because reliable national cost data are available for lung cancer in Colombia [15]. Although GBD also reports data for other definitively asbestos-related malignancies (laryngeal and ovarian cancer), the absence of condition-specific cost estimates in the Colombian context limits their inclusion at this stage of analysis.

In Colombia, industrial asbestos use began in 1942 and continued for several decades through the manufacture of asbestos cement products, automotive components [16], and other industrial applications before the comprehensive ban established under Law 1968 of 2019 [17]. While this legislation represents a major public health achievement, the long latency period of asbestos-related diseases means that the burden arising from historical exposure will continue for decades. According to the Global Burden of Disease estimates for 2022 [18], Colombia had 7929 prevalent cases of lung cancer from all causes and 150 prevalent cases of mesothelioma. In addition, a recent national mortality study documented 1539 mesothelioma deaths between 1997 and 2022 and identified substantial diagnostic and surveillance limitations, suggesting that the true burden of asbestos-related disease may be underestimated [19].

Cost-of-illness (COI) studies provide a framework for quantifying the economic burden imposed by specific diseases on healthcare systems and society [13]. These analyses typically distinguish between direct medical costs, indirect costs related to productivity losses, and, in some cases, losses associated with diminished quality of life. In the specific context of asbestos-related diseases, COI approaches have previously been applied to estimate direct and societal costs from a social perspective. In Colombia, direct costs attributable to lung cancer for the health system have previously been estimated for 2017 using national administrative databases and propensity score matching techniques [15]. That study provides a valuable foundation for understanding the economic burden of lung cancer in Colombia and serves as the basis for the direct cost estimates used in the present analysis. Building on this evidence, the current study focuses on a different question: the economic burden attributable to asbestos-related malignancies. To address this objective, updated epidemiological estimates and a broader societal perspective were incorporated into the analytical framework.

The absence of a comprehensive asbestos-attributable economic estimate limits a full understanding of the current health and financial impact of historical exposure in Colombia. Quantifying this burden is essential not only for assessing present economic implications but also for supporting evidence-based policymaking in areas such as epidemiological surveillance, disease registry strengthening, health system planning, and resource allocation. Importantly, these estimates also highlight the potential long-term economic consequences of inadequate control of ongoing asbestos exposure, particularly from asbestos remaining in place within existing buildings and from unsafe disturbance or removal practices [20,21].

Therefore, the objective of this study is to estimate the annual economic impact attributable to asbestos exposure in Colombia for lung cancer and mesothelioma in 2022. A societal cost-of-illness approach is adopted, integrating updated national direct medical cost data, GBD epidemiological estimates, and progressive scenarios incorporating indirect costs and monetized quality-of-life losses. Each cost component is modeled separately to avoid double counting and ensure internal consistency of the analytical framework.

## 2. Materials and Methods

### 2.1. Study Design and Analytical Framework

A prevalence-based cost-of-illness (COI) study was conducted to estimate the annual economic burden attributable to asbestos exposure in Colombia for lung cancer and mesothelioma in 2022. The analysis was performed from a societal perspective, integrating direct medical costs, indirect costs due to premature mortality, and monetized quality-of-life losses. Two attribution approaches were used to estimate the asbestos-attributable burden of lung cancer: (1) a population-attributable fraction (PAF) approach derived from GBD risk modeling and (2) a secondary 1:3 mesothelioma-to-lung cancer ratio scenario used as a structural sensitivity analysis.

A prevalence-based annual approach was selected because the only available nationally validated cost data corresponds to total annual healthcare expenditures for prevalent cases of lung cancer in 2017 [15]. Consequently, direct costs were modeled using an annual-prevalent framework rather than an incidence–lifetime approach. All monetary values are reported as inflation-adjusted 2022 values derived from the original 2017 PPP-adjusted international dollar estimates.

### 2.2. Data Sources

#### 2.2.1. Epidemiological Data

Epidemiological estimates were obtained from the Institute for Health Metrics and Evaluation (IHME) Global Burden of Disease (GBD) Results Tool using the 2022 estimates for Colombia [18]. Lung cancer prevalence, mesothelioma prevalence, mortality, and disability-adjusted life years (DALYs) were extracted from the GBD Results Tool. Population-attributable fractions (PAFs) for occupational asbestos exposure were obtained from GBD Compare using the corresponding 2022 estimates available through the IHME visualization platform. Data were extracted in March 2026 and were based on national estimates for both sexes and all ages.

The GBD 2022 prevalence estimates used in the analysis were 7929 prevalent cases of lung cancer and 150 prevalent cases of mesothelioma. GBD 2017 prevalence estimates were used to derive an implied annual direct cost per prevalent lung cancer case from the 2017 Colombian cost-of-illness study. Administrative registry data from the Colombian High-Cost Account (Cuenta de Alto Costo, CAC) were used for contextual comparison but not as the primary source of epidemiological burden [22], given that CAC reflects insurance-based reporting within the health system rather than population-level modeling and may not fully capture the population burden of disease.

#### 2.2.2. Direct Cost Data

Direct medical costs were derived from a nationally published cost-of-illness study conducted in Colombia for 2017 [15]. That study identified 5387 prevalent lung cancer cases and estimated total attributable direct costs between USD 50,039,588 and USD 74,468,111 (2017 international dollars, adjusted for purchasing power parity [PPP]).

The monetary values reported in the original study were expressed as 2017 international dollars adjusted for purchasing power parity (PPP). In the present analysis, these values were not converted to nominal U.S. dollars. Instead, they were updated to the 2022 base year by applying the official Colombian Consumer Price Index (CPI) published by DANE. Accordingly, all monetary values reported in this study represent inflation-adjusted updates of the original PPP-based estimates, preserving the monetary framework of the source study while expressing the results in 2022 price levels.

The implied annual cost per prevalent case in 2017 was calculated as

$${IC}_{2017}=\frac{{TC}_{2017}}{Prev_{2017}}$$

where ${TC}_{2017}\;$is the total direct cost, and ${Prev}_{2017\;}$ is the number of prevalent lung cancer cases. This yielded an estimated per-patient annual direct cost range.

#### 2.2.3. Inflation Adjustment

Costs were updated from 2017 to 2022 using the official Consumer Price Index (CPI) published by the Colombian National Administrative Department of Statistics (DANE) [23].

The cumulative inflation factor between 2017 and 2022 was calculated as follows:

$$Factor\left(2017\to 2022\right)\;=\left(1+\pi 2018\right)\left(1+\pi 2019\right)\left(1+\pi 2020\right)\left(1+\pi 2021\right)\left(1+\pi 2022\right)=1.293$$

where $\pi_{t}\;$represents annual CPI variation.

The updated per-patient direct cost in 2022 was, therefore,

c2022=c2017×Factor(2017→2022)

### 2.3. Estimation of Direct Medical Costs in 2022

Because the original cost study was prevalence-based, total direct costs in 2022 were estimated by multiplying the updated per-case cost by 2022 GBD point prevalence:

For lung cancer:

DirectCost(LC,2022)=c(LC,2022)×7929

For mesothelioma, no Colombia-specific cost-of-illness estimate was identified. Therefore, the annual per-case cost of lung cancer was used as a pragmatic proxy. This assumption does not imply clinical or therapeutic equivalence between the two diseases. To reflect uncertainty in transferring costs across malignancies, the mesothelioma proxy was varied by ±20% in deterministic sensitivity analysis. Because no empirical Colombian data were available to estimate the relative cost differential, the direction and magnitude of the potential transfer bias could not be determined a priori.

### 2.4. Attribution to Asbestos Exposure

Mesothelioma was considered predominantly attributable to asbestos exposure. Therefore, the attributable fraction (AF) for mesothelioma was set at approximately 1.0 (100%), consistent with the well-established causal association between asbestos exposure and mesothelioma.

For lung cancer, only a fraction of cases is attributable to asbestos exposure. Therefore, population-attributable fractions (PAFs) derived from GBD risk modeling were applied. PAF estimates for occupational asbestos exposure were obtained from GBD Compare using the corresponding 2022 estimates available through the IHME visualization platform. For Colombia in 2022, the PAF for lung cancer attributable to occupational asbestos exposure was 4.9%, as reported by the GBD risk-attribution framework.

The PAF was applied to both prevalence and mortality estimates in order to derive the fraction of lung cancer burden attributable to occupational asbestos exposure. The asbestos-attributable prevalence for lung cancer was calculated as

$$Prev\left(LC,attrib\right)=PrevLC\times PAF\;asbestos$$

Similarly, asbestos-attributable mortality was estimated as

$$Deaths\left(LC,attrib\right)=DeathsLC\times PAF\;asbestos$$

Direct costs attributable to asbestos exposure were estimated by applying the asbestos-attributable fraction to lung cancer direct costs and adding mesothelioma direct costs, which were considered fully attributable to asbestos exposure.

$$DirectCost\;attrib=DirectCost\left(LC,2022\right)\times PAF\;asbestos+DirectCost\;\left(Meso,2022\right)$$

### 2.5. Indirect Costs (Productivity Losses)

Indirect costs were estimated using the human capital approach, calculating the present value of productivity losses associated with premature mortality attributable to asbestos-related disease.

The general expression was as follows:

$$Indirect\;Cost=\sum \left(Deaths\;attrib\times PV\left(Earnings\right)\right)$$

where $Deaths_{attrib}$ represents asbestos-attributable deaths, and $PV\left(Earnings\right)\;$represents the discounted present value of expected future earnings.

Future earnings were estimated using the Colombian minimum annual wage plus the statutory transportation allowance for 2022. A conservative assumption of 10 years of potential working life lost per premature death and a 3% annual discount rate were applied to estimate productivity losses. Indirect costs were reported separately from direct medical costs to avoid double counting.

### 2.6. Monetization of Quality-of-Life Losses

Quality-of-life losses were estimated using GBD-derived disability and mortality components (DALYs) and converted into monetary values using alternative willingness-to-pay (WTP) thresholds based on multiples of Colombia’s 2022 GDP per capita (1× and 3× GDP per capita) [24].

The general expression was as follows:

DALYCost=DALYlost×WTPthreshold

Because productivity losses may partially overlap with the monetization of DALYs, this component was reported separately within the expanded societal cost scenarios.

### 2.7. Scenario Analysis

Three progressively inclusive scenarios were constructed:

1. Direct medical costs only;
2. Direct costs + productivity losses;
3. Direct costs + productivity + DALY valuation.

In addition, sensitivity analyses were performed by varying key parameters, including the population-attributable fraction (PAF), discount rate, and mesothelioma cost proxy assumptions. This structured approach ensured internal consistency and avoided mixing incompatible epidemiological measures (e.g., applying mortality counts to prevalence-based costs).

The deterministic sensitivity analysis adopted in this study was selected because the principal Colombian epidemiological and economic inputs were available only as published point estimates, without empirical probability distributions or variance parameters required for probabilistic sensitivity analysis. Under these circumstances, deterministic scenario analysis was considered the most transparent and reproducible approach for evaluating structural uncertainty while avoiding unsupported assumptions regarding parameter distributions. The principal assumptions underlying the economic model are summarized in Table 1.

## 3. Results

### 3.1. Epidemiological Inputs (GBD)

For 2022, GBD estimates for Colombia reported 150 prevalent cases of mesothelioma and 7929 prevalent cases of lung cancer. Application of the GBD occupational asbestos-attributable fraction (4.9%) yielded an estimated 389 asbestos-attributable lung cancer cases. Under the alternative 1:3 mesothelioma-to-lung-cancer ratio scenario, the estimated number of asbestos-attributable lung cancer cases was 450 (Table 2).

### 3.2. Direct Annual Unit Cost

Based on the Colombian cost-of-illness study for lung cancer, the estimated annual direct cost per prevalent case in 2017 ranged from USD 9285 to USD 13,827 [15]. After adjustment for inflation to 2022 values, the corresponding annual direct cost ranged from USD 12,007 to USD 17,882 per case-year. In the absence of Colombia-specific cost estimates for mesothelioma, this range was used as a proxy estimate for mesothelioma-related direct costs, with sensitivity analyses assuming ±20% variation.

### 3.3. Asbestos-Attributable Direct Costs in 2022

#### 3.3.1. Scenario A: PAF-Based Attribution

Under the PAF-based attribution approach, direct costs attributable to asbestos-related lung cancer were estimated at USD 4.67–6.96 million, while direct costs attributable to mesothelioma were estimated at USD 1.80–2.68 million. Total direct costs attributable to asbestos-related malignancies ranged from USD 6.47 to 9.64 million in 2022.

#### 3.3.2. Scenario B: 1:3 Mesothelioma-to-Lung-Cancer Ratio

Under the alternative 1:3 attribution scenario, direct costs attributable to lung cancer were estimated at USD 5.40–8.05 million, while mesothelioma-related direct costs remained between USD 1.80 and 2.68 million. Total direct costs attributable to asbestos-related malignancies ranged from USD 7.20 to 10.73 million. Despite the different attribution assumptions, both approaches produced broadly similar estimates of direct costs attributable to asbestos-related malignancies (Table 3).

### 3.4. Indirect Costs Attributable to Asbestos in 2022

#### 3.4.1. Attributable Mortality

For the PAF-based scenario, GBD estimates indicated 265 lung cancer deaths attributable to occupational asbestos exposure and 115 mesothelioma deaths, resulting in a total of 380 asbestos-attributable deaths in 2022. Under the alternative 1:3 mesothelioma-to-lung-cancer ratio scenario, the estimated number of asbestos-attributable lung cancer deaths was 345, resulting in a total of 460 asbestos-attributable deaths.

#### 3.4.2. Productivity Losses

Using the human capital approach described in the Methods section, indirect costs attributable to premature mortality were estimated at USD 10.21 million under the PAF scenario and USD 12.36 million under the alternative 1:3 scenario (Table 4).

### 3.5. Direct and Indirect Costs Attributable to Asbestos

Under the PAF-based attribution scenario, total costs attributable to asbestos-related malignancies ranged from USD 16.68 to 19.85 million, including direct costs of USD 6.47–9.64 million and productivity losses of USD 10.21 million. Under the alternative 1:3 mesothelioma-to-lung-cancer ratio scenario, total costs ranged from USD 19.56 to 23.09 million, including direct costs of USD 7.20–10.73 million and productivity losses of USD 12.36 million (Table 5).

### 3.6. Quality-of-Life Losses Attributable to Asbestos

GBD estimates indicated 3080 DALYs associated with mesothelioma and 5044.16 DALYs attributable to occupational asbestos-related lung cancer in Colombia during 2022. The total burden attributable to asbestos-related malignancies was therefore estimated at 8124.16 DALYs. Using a willingness-to-pay threshold equivalent to one times Colombia’s GDP per capita, the monetized value of DALY losses was estimated at USD 54.27 million. Using a threshold equivalent to three times GDP per capita, the corresponding estimate increased to USD 162.82 million.

### 3.7. Expanded Societal Valuation Scenarios

The expanded societal scenarios presented below should be interpreted as complementary valuation scenarios rather than as a single additive estimate of economic cost. Although direct medical costs, productivity losses, and monetized DALY losses are presented together to illustrate progressively broader perspectives of societal burden, productivity losses and DALY monetization may partially capture overlapping consequences of premature mortality and disability. Consequently, these scenarios are intended to inform complementary perspectives on the economic and societal impact of asbestos-related diseases rather than to represent a single definitive estimate of total economic cost (Table 6).

## 4. Discussion

This study provides a comprehensive estimate of the annual economic burden attributable to asbestos exposure in Colombia, integrating direct medical costs, productivity losses due to premature mortality, and monetized quality-of-life losses. The analysis combines nationally derived cost data with Global Burden of Disease (GBD) epidemiological estimates to quantify the persistent health and economic consequences associated with historical asbestos exposure in a post-ban setting.

The results demonstrate that the direct healthcare burden attributable to asbestos-related malignancies in 2022 ranged between approximately USD 6 and 11 million annually, depending on the attribution scenario applied. When productivity losses due to premature mortality were incorporated, the estimated burden increased to approximately USD 17–23 million annually. The largest increase in magnitude was observed when DALY-based quality-of-life losses were monetized.

These findings indicate that even under the most conservative scenario, direct costs attributable to asbestos-related malignancies exceeded USD 6 million annually, while broader societal estimates approached USD 180 million per year.

Under a valuation threshold equivalent to one time Colombia’s GDP per capita per DALY, the total societal burden was estimated at approximately USD 71–74 million. Under a threshold equivalent to three times GDP per capita per DALY, the estimated societal burden exceeded USD 180 million annually.

Importantly, both attribution approaches—the PAF-based estimation derived from GBD occupational asbestos exposure estimates and the alternative 1:3 mesothelioma-to-lung-cancer ratio—produced relatively similar economic estimates. This consistency reflects the similarity of the underlying epidemiological assumptions used in both approaches and suggests that the estimated economic burden remains substantial across alternative attribution frameworks.

Although direct comparisons are challenging because methodologies, attribution approaches, and healthcare systems differ across countries, previous studies from Canada have similarly reported substantial societal costs associated with asbestos exposure [13]. The magnitude of the burden estimated in the present study is consistent with these observations and reinforces the importance of considering societal costs when evaluating the long-term consequences of asbestos exposure.

These findings may also have broader relevance for other low- and middle-income countries where historical asbestos use has occurred, but disease-specific economic evidence remains scarce. Similar to Colombia, many of these countries rely on incomplete surveillance systems, limited occupational disease registries, and fragmented health economic information. Under these circumstances, transparent cost-of-illness frameworks based on the best available national evidence can provide an initial basis for policy development, priority setting, and resource allocation while highlighting critical evidence gaps that require future investigation.

A major strength of this analysis is the integration of updated epidemiological volumes, inflation-adjusted national cost estimates, and broader societal cost components within a unified analytical framework. Previous Colombian cost-of-illness studies have provided important estimates of direct healthcare expenditures associated with lung cancer and constitute the methodological foundation upon which the present analysis builds. The current study extends that evidence by applying updated epidemiological estimates and a broader societal perspective to estimate the economic burden attributable specifically to asbestos-related malignancies. By incorporating these additional components, the present analysis provides a broader estimate of the societal implications associated with asbestos-related malignancies in Colombia.

The study also highlights important structural differences between population-modeled epidemiological estimates and administrative healthcare reporting systems. The discrepancies observed between GBD estimates and Colombian High-Cost Account (CAC) registry counts likely reflect differences in case ascertainment, reporting completeness, and methodological approach. Because CAC is based on insurance-related reporting within the healthcare system rather than population-level modeling, it may not fully capture the population burden of disease. These findings reinforce the importance of strengthening epidemiological surveillance systems and improving the completeness of asbestos-related disease reporting.

Another important implication of this work is that it highlights the long-term economic consequences of inadequate control of ongoing asbestos exposure. Although asbestos has been legally banned in Colombia since 2019, installed asbestos remains widely present in buildings, industrial infrastructure, and residual materials. Unsafe removal practices, demolition activities, informal waste handling, and other uncontrolled interventions may continue generating environmental and occupational exposure pathways for decades. Consequently, the burden estimated in this study should not be interpreted solely as a historical legacy phenomenon but also as evidence of the potential future costs associated with inadequate management of installed asbestos.

Several limitations should be acknowledged. First, Colombia-specific direct cost estimates for mesothelioma were unavailable. Therefore, lung cancer direct costs were used as a proxy estimate for mesothelioma, accompanied by sensitivity analyses to reflect uncertainty around this assumption. Second, the productivity-loss estimates relied on simplified assumptions regarding wage levels and years of potential working life lost. Third, DALY monetization inherently depends on normative willingness-to-pay assumptions, which may vary across economic contexts.

The human capital approach adopted in this study also has inherent methodological limitations. By valuing productivity losses on the basis of potential future earnings, this approach may not fully capture broader societal impacts or alternative economic perspectives, such as those represented by the friction cost approach. Nevertheless, the human capital method remains one of the most widely used approaches in cost-of-illness studies and was considered appropriate for the objectives of this prevalence-based cost-of-illness study and the availability of Colombian epidemiological and economic data.

The use of lung cancer costs as a proxy for mesothelioma may result in either underestimation or overestimation of disease-specific expenditures. Mesothelioma may involve complex diagnostic pathways, multimodal treatment, major thoracic surgery, systemic therapy, and specialized supportive care, which could make the proxy conservative in some clinical settings. Conversely, differences in treatment eligibility, disease progression, survival, and actual access to specialized therapies may reduce resource utilization for some patients. In the absence of Colombian patient-level costing data, the net direction and magnitude of this bias remain uncertain. More broadly, this limitation reflects the scarcity of disease-specific economic evidence for rare occupational cancers in Colombia, highlighting an important evidence gap rather than a limitation unique to the present study.

The deterministic sensitivity analysis adopted in this study also deserves consideration. Because the available Colombian epidemiological and economic sources report point estimates rather than empirical probability distributions or variance parameters, probabilistic sensitivity analysis could not be implemented without introducing additional distributional assumptions. Under these circumstances, deterministic scenario analysis was considered the most transparent and reproducible approach for evaluating structural uncertainty. Future studies based on patient-level costing data or richer epidemiological datasets may allow formal probabilistic modeling of uncertainty.

Another limitation concerns the inflation adjustment applied to direct medical costs. Because no disease-specific or nationally validated healthcare cost deflator was available for the costing framework used in this study, costs were updated using the official Consumer Price Index (CPI) published by DANE. General CPI may not fully capture price dynamics affecting specialized medical care, including pharmaceuticals, advanced technologies, hospital services, and oncology treatments. Consequently, the updated direct costs presented here may represent conservative estimates of the current economic burden. Future studies should evaluate alternative healthcare-specific inflation measures as more detailed Colombian cost indices become available.

A further methodological consideration concerns the interpretation of the expanded societal scenarios. Although the potential overlap between productivity losses and DALY monetization was explicitly acknowledged in the analytical framework, these scenarios were intended to represent complementary valuation perspectives rather than strictly additive estimates of economic burden. Future methodological developments could further refine approaches for integrating these components while minimizing conceptual overlap.

The attribution framework used by the Global Burden of Disease study also introduces important limitations. The GBD-derived attributable fractions and lung cancer DALY estimates used in this analysis refer specifically to occupational asbestos exposure and do not fully capture environmental, paraoccupational, or other non-occupational asbestos exposure pathways. In contrast, the mesothelioma component incorporated all-cause mesothelioma burden estimates because mesothelioma is strongly associated with asbestos exposure beyond strictly occupational settings.

Consequently, the estimates presented here may underestimate the broader asbestos-related burden associated with installed asbestos, environmental contamination, or community exposure. Future studies should evaluate additional asbestos-related malignancies, incorporate Colombia-specific mesothelioma cost data, and explore the economic burden associated with environmental and paraoccupational asbestos exposure.

Despite these limitations, the integration of national cost data, official inflation indices, GBD epidemiological estimates, and expanded societal-cost scenarios provides a transparent, reproducible, and policy-relevant framework for estimating the economic burden of asbestos-related diseases in middle-income settings where disease-specific economic evidence remains limited. The findings support the need for strengthened epidemiological surveillance, improved disease registries and reporting systems, long-term management of installed asbestos, and broader preventive policies aimed at reducing future asbestos-related disease burden in Colombia.

## 5. Conclusions

Asbestos-related lung cancer and mesothelioma continue to impose a substantial economic burden in Colombia despite the national asbestos ban enacted in 2019. Depending on the attribution and valuation approach applied, expanded societal valuation scenarios for asbestos-related malignancies ranged from approximately USD 71 million to more than USD 180 million. These findings demonstrate that the economic consequences of asbestos exposure remain important due to the prolonged latency period of asbestos-related diseases and the persistence of installed asbestos throughout the country.

Beyond the direct economic implications, these findings have important public health and policy relevance. The results suggest that the burden associated with asbestos exposure is not limited to historical occupational exposure alone. Installed asbestos, unsafe removal practices, demolition activities, informal waste handling, and environmental contamination may continue generating exposure pathways and future disease burden for decades.

The study demonstrates the importance of incorporating broader societal costs into asbestos-related disease assessments, particularly in middle-income countries where the long-term economic consequences of asbestos exposure remain insufficiently quantified. The findings support the need for strengthened surveillance systems, improved disease registries, and long-term strategies for the safe management of installed asbestos to reduce future health and economic burdens.

## Figures and Tables

**Table 1:** Key assumptions underlying the economic model.

Component	Assumption	Justification/Source
Study design	Prevalence-based cost-of-illness	National annual cost data available
Base year	2022	GBD estimates
Perspective	Societal	Study design
Mesothelioma attributable fraction	Approximately 100%	Established causal association with asbestos (IARC)
Lung cancer attributable fraction	4.9%	GBD Compare (occupational asbestos exposure)
Mesothelioma direct cost	Lung cancer cost used as proxy	No Colombian mesothelioma cost study available
Inflation adjustment	General CPI (DANE)	Official national inflation index
Productivity losses	Human Capital Approach	Standard COI methodology
Working life lost	10 years	Conservative assumption
Discount rate	3%	Standard economic evaluation
DALY valuation	1× and 3× GDP per capita	Alternative WTP scenarios
Sensitivity analysis	Deterministic	No empirical parameter distributions available

**Table 2:** Epidemiological burden of asbestos-related malignancies in Colombia, 2022.

Disease/Scenario	Attributable Prevalent Cases
Mesothelioma (AF ≈ 1)	150
Lung cancer (PAF 4.9%)	389
Lung cancer (1:3 scenario)	450

**Table 3:** Direct costs attributable to asbestos-related malignancies in Colombia, 2022.

Disease/Scenario	Lower Estimate (USD Million)	Upper Estimate (USD Million)
Lung cancer (PAF)	4.67	6.96
Mesothelioma (PAF)	1.80	2.68
Total (PAF)	6.47	9.64
Lung cancer (1:3)	5.40	8.05
Mesothelioma (1:3)	1.80	2.68
Total (1:3)	7.20	10.73

**Table 4:** Attributable mortality and indirect costs associated with asbestos-related malignancies in Colombia, 2022.

Scenario	Attributable Deaths	Indirect Costs (USD Million)
PAF-based attribution	380	10.21
1:3 mesothelioma-to-lung-cancer ratio	460	12.36

**Table 5:** Direct and indirect costs attributable to asbestos-related malignancies in Colombia, 2022.

Scenario	Direct Costs (USD Million)	Indirect Costs (USD Million)	Total Costs (USD Million)
PAF (lower estimate)	6.47	10.21	16.68
PAF (upper estimate)	9.64	10.21	19.85
1:3 (lower estimate)	7.20	12.36	19.56
1:3 (upper estimate)	10.73	12.36	23.09

**Table 6:** Expanded Societal Valuation Scenarios attributable to asbestos exposure in Colombia, 2022.

Scenario	Direct Costs (USD Million)	Indirect Costs (USD Million)	DALY Valuation (USD Million)	Societal Valuation Scenario (USD Million)
PAF–1× GDPpc	6.47–9.64	10.21	54.27	70.95–74.12
PAF–3× GDPpc	6.47–9.64	10.21	162.82	179.50–182.67
1:3–3× GDPpc	7.20–10.73	12.36	162.82	182.38–185.91

## Data Availability

All data used in this study were obtained from publicly available sources, including the Global Burden of Disease (GBD) Results Tool, the Colombian National Administrative Department of Statistics (DANE), and World Bank databases. No individual-level data were used in this analysis.
